# Wing Morphometry and Acoustic Signals in Sterile and Wild Males: Implications for Mating Success in *Ceratitis capitata*

**DOI:** 10.1155/2015/526969

**Published:** 2015-05-14

**Authors:** João Maria Gomes Alencar de Souza, Paulo Augusto de Lima-Filho, Wagner Franco Molina, Lúcia Maria de Almeida, Milson Bezerra de Gouveia, Francisco Pepino de Macêdo, Raul Alberto Laumann, Beatriz Aguiar Jordão Paranhos

**Affiliations:** ^1^Department of Cellular Biology and Genetics, Bioscience Center, Federal University of Rio Grande do Norte, Campus Universitário, 59078-970 Natal, RN, Brazil; ^2^Federal Institute os Science and Tecnology of Rio Grande do Norte, 59500-000 Macau, RN, Brazil; ^3^Brazilian Agricultural Research Corporation (EMBRAPA), Embrapa Genetic Resources and Biotechnology, Pq. Est. Biol., Final W5 Norte, 70770-917 Brasília, Brazil; ^4^Brazilian Agricultural Research Corporation (EMBRAPA), Embrapa Tropical Semi-arid, BR 428, km 152, 56302-970 Petrolina, PE, Brazil

## Abstract

The sterile insect technique (SIT) is widely utilized in the biological control of fruit flies of the family Tephritidae, particularly against the Mediterranean fruit fly. This study investigated the interaction between mating success and morphometric variation in the wings and the production of acoustic signals among three male groups of *Ceratitis capitata* (Wiedemann): (1) wild males, (2) irradiated with Co-60 (steriles), and (3) irradiated (steriles) and treated with ginger oil. The canonical variate analysis discriminated two groups (males irradiated and males wild), based on the morphological shape of the wings. Among males that emit buzz signals, wild males obtained copulation more frequently than males in Groups 2 and 3. The individuals of Group 3 achieved more matings than those in Group 2. Wild males displayed lower pulse duration, higher intervals between pulses, and higher dominant frequency. Regarding the reproductive success, the morphological differences in the wings' shape between accepted and nonaccepted males are higher in wild males than in the irradiated ones. The present results can be useful in programs using the sterile insect technique for biological control of *C. capitata*.

## 1. Introduction

True fruit flies (Diptera: Tephritidae) are well known worldwide because they infest economically important fruits. In the family, the Mediterranean fruit fly* Ceratitis capitata* (Wiedemann) is considered one of the most important pests of fruit crops worldwide. Currently, one of the most widely used techniques in controlling* C. capitata* is the sterile insect technique (SIT), considered a type of autocidal or genetic control, in which the pest is utilized for its own control. Sterile males are released in production areas where they will compete with the wild males to copulate with wild females [1].

In tephritids, such as* C. capitata*, the mating compatibility between the sterile male and the wild female is problematic, because the species displays a lek mating system characterized by active female choice. In male aggregations, at least five males compete for copulations [2, 3]. When the female approaches the lek, males begin the cohort ritual involving multimodal signals consisting of movements with the head (visual signals), vibration of the wings (sound signals), and pheromone release (chemical signals). In these aggregations, males start the communication with a “calling song” characterized by a continuous vibration of wings [4, 5] at fundamental frequency of about 350 Hz [6]. During the calling song, males bent their abdomen ventrally everting the rectal epithelium and producing a drop of pheromone. The simultaneous wing vibration and abdominal curvature presumably serve to direct the pheromone plume directly at the female. Upon female approach, the male typically begins courtship display that is characterized by intermittent emission of wing buzzes with a fundamental frequency of 165 Hz; each of these pulses has a mean duration of 100 ms [7]. Males emit these signals until they leap onto the female or until they are rejected. The courtship song is accompanied by visual displays of the males including the head rapidly rocking from side to side and usually tapping the female with their aristae [5].

If the female is receptive, the male rides it and tries to assure copulation. Females are sometimes receptive to some males' cohort; however, they accept only one sexual partner [8]. On the other hand, the female may abandon the lek at any time, and sometimes, despite accepting initially a male as a sexual partner, the female may further refuse it. When released in the field, the sterile males of* C. capitata* should be capable of joining or forming leks in the host plant, attracting wild females and copulating with them [1].

Participation in leks is the first step that the released males must successfully accomplish in order to have the chance to obtain mating success. These males must exhibit a behavioral repertoire that closely matches that of wild individuals, because wild females of* C. capitata* are more receptive to wild males than to sterile ones or males originated from mass rearing [9, 10]. Therefore, any deviation in the expression of wild sexual behavior, even small one, may substantially reduce males reproductive competitiveness in the field and could reduce effectiveness of SIT. In addition, the mass production, independent of irradiation, reduces the ability of* C. capitata* males to induce wild females to copulate [11]. Therefore, recent research efforts have focused on finding simple but effective ways to improve the quality of adult fruit flies destined for field release in SIT programs worldwide. Recent studies have shown that aromatherapy of sterile males with ginger root oil (GRO) has improved mating performance [12–16].

Can the female of* C. capitata* detect some characteristics in a male that make it attractive as a sexual partner? This issue was highlighted by several authors who sought an answer to this question by analyzing behavioral and morphometric characters in males [3, 17, 18]. Thus, Hasson and Rossler [17] questioned, for example, the influence of fluctuating asymmetry (FA) of morphological characters acting in sexual selection, with symmetrical males potentially achieving greater mating success than asymmetrical males. In fact, in* C. capitata*, there is a positive correlation between mating success and symmetry of frontal supraorbital bristles. It is also known that the stress suffered during development due to the high larval density on the process of* C. capitata* mass production can contribute to increasing morphological asymmetry in structures with bilateral symmetry [3]. Souza et al. [19] observed in* Anastrepha zenildae* that males with symmetrical distribution of the bristles on the front plate achieved greater success in mating than asymmetrical males.

Geometric morphometrics is a relatively new technique that is employed for the analysis of the variation in shape [20, 21]. The structures' shape measures have proven to be more informative than the linear dimensions, in addition to offering an extremely high power to detect very subtle phenotypic variations [22].

By using the technique of geometric morphometrics, this study investigated the shape variation of wings between the groups of wild* C. capitata* males and those irradiated with gamma radiation and its relationship with the production of acoustic signals in order to define whether possible morphological alterations may influence the acoustic communication and male mating success.

## 2. Material and Methods

Adult wild individuals of* C. capitata* were obtained from infested fruits of guava (*Psidium guajava*), which were collected in orchards located in the region of Petrolina, São Francisco River Valley, in Northeastern Brazil (23′39′′S, 40°30′35′′W). After sampling, fruits were taken to the Semi-arid Entomology Laboratory of Embrapa and placed in plastic trays containing vermiculite as substrate and remained there until specimens of* C. capitata* completed their larval development. After 10–15 days, the vermiculite was sifted to obtain the pupae which were later transferred and kept in acrylic boxes (30 cm cubes) until the emergence of adults. Then, the flies were separated by sex and kept separately in net cages (30 cm cubes), under natural conditions, with temperature ranging between 27–31°C and relative humidity between 10–80%, with water and an* ad libitum* diet (sucrose : hydrolyzed protein, 3 : 1) until they reached sexual maturity, which occurred between 8–10 days after adult emergence.

Sterile males, mutant strain* tsl*-Vienna 8, were supplied by Biofábrica Moscamed Brasil located in Juazeiro, Bahia state, Northeastern Brazil. Males were irradiated during the pupal stage, with a dose of 95 Gy of gamma radiation (Co-60), two days before the emergence of adults. The irradiated pupae were then divided into two net cages (30 cm cubes), each containing 40 mL of pupae. Both cages were provided with artificial diet (3 sugar : 1 hydrolyzed protein) and water* ad libitum* and kept on a controlled room (25°C ± 2°C; 55 ± 10% of humidity). In the 4th to 5th day after emergence, the flies from one of the cages were exposed to 1.5 mL of ginger root oil of* Zingiber officinale* Roscoe (Zingiberaceae) (GRO) in a small room (27 m^3^) for 20 hours, with 25–27°C, 50–60% RH, and 12 h photoperiod (6:30–18:30 h), where the GRO aroma was dispersed by air fans [23]. The cage with untreated males was kept in a separate room.

One day before the beginning of sexual compatibility tests, wild males and females (8–15 days old) and the GRO-treated and nontreated sterile males (5–7 days old) were marked on the thorax with a small drop of nontoxic and water-soluble white ink (Faber Castell), by alternating the strain that was painted, in order to distinguish between them. To this end, ten individuals at a time were placed inside tulle bags, as they were immobilized between two fingers, one by one, in order to make the painting easier.

Mating tests were conducted in four field cages measuring 2 × 2 × 2 m, located in the shade under tree canopies. Each field cage contained a* Ficus* spp. plant inside, measuring 1.80 m (height), with enough foliage to house groups of calling males. In each cage, ten wild males and ten sterile males were released at 7:30 a.m. Wild males and sterile males were released into two cages and wild and sterile GRO-treated males were released into two other cages. After males formed leks and initiated calling behavior, 10 virgin wild females were released into each cage. The mating couples were collected from 7:45 a.m. until 12:30 p.m. and kept separately in transparent acrylic tubes until the end of copulation. Then, males, whether they are successful or not in mating, were individualized in Eppendorf tubes (2 mL), forming six groups: accepted and not accepted wild males for copulation, accepted and not accepted sterile males, and accepted and not accepted sterile males treated with GRO.

### 2.1. Analysis of Geometric Morphometrics

**Figure 1:**
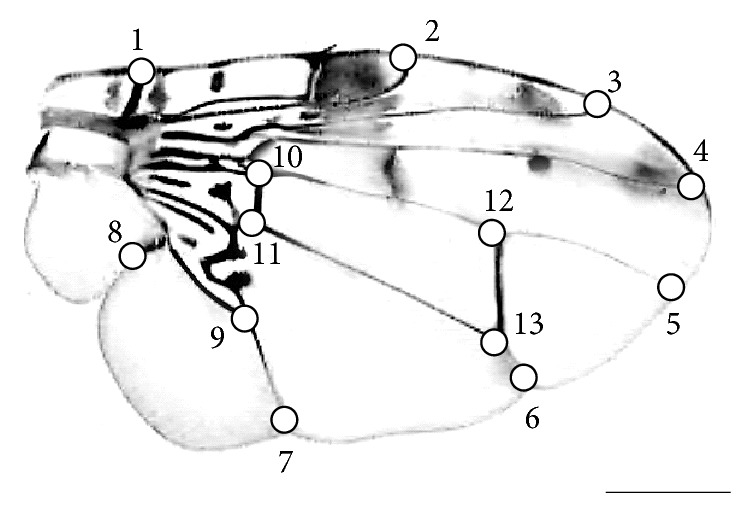
Landmarks in the wing of* Ceratitis capitata*. 1: intersection between the humeral and costal veins; 2: intersection between the radial R_1_ and costal veins; 3: intersection between R_2+3_ and costal veins; 4: intersection between R_4+5_ and costal veins; 5: intersection between M and apical marginal veins; 6: intersection between the anterior cubital CuA_1_ and marginal apical veins; 7: intersection between the anterior cubital CuA_2_ and marginal posterior veins; 8: intersection between Cu_2+_A veins; 9: intersection between cubital M CuA_1_ and median cubital basal veins; 10: intersection between CuA_1_ and midcubital distal veins; 11: intersection between r-m and R_4+5_ veins; 12: intersection between r-m and M veins; 13: intersection between the dm-cu and CuA_1_ veins.

In order to perform the analysis by geometric morphometrics, 50 males free of injury, from each category of males selected in the experiment described above, had their left wing gently removed and then mounted between a slide and a coverslip with the use of cedar oil. The scanned images of these structures were obtained from a digital camera Motic 5.0 attached to a stereomicroscope. The TpUtil software was used to randomize the images and subsequently Tps Dig [24] was used to identify thirteen landmarks (Figure 1). Information on the shapes of landmark coordinates by the analysis of Procrustes superimposition [21] was obtained through MorphoJ 1.02b software [25]. Procrustes distances (Prc dist), Mahalanobis distances (*D* ^2^), and Canonical Variables Analysis (CVA) were performed in order to detect variables capable of distinguishing individuals, and from this analysis, MANOVA, comparative deformation grids were obtained from the comparison of CV1. Warped outlines were generated from the landmark coordinates to more clearly demonstrate the vector variations of deformation grids related to the analysis of each group of* C. capitata* which consisted of wild males (Group 1), irradiated sterile males (Group 2), and irradiated sterile males aromatically treated with ginger oil (GRO) (Group 3), with and without successful copulation.

### 2.2. Acoustic Communication: Recording Procedures and Analyses

An experimental arena was constructed with a cylindrical plastic cage (18 cm diameter × 5 cm height). The microphone used for recording was inserted through a small hole (3.5 cm diameter) on the top of the cage.

Males (Groups 1, 2, and 3) and females with 10–20 days old were introduced into the arena. In general, the experiment was initiated with 3–5 males and 1 female, and more insects were introduced to a maximum of 10 males and 6 females. If the insects were nonresponsive after 10 minutes of observation, they were replaced by new ones.

Experiments were conducted at Embrapa Genetic Resources and Biotechnology (Brasília, DF, Brazil) in a sound-insulated room on a shock-proof table in order to decrease environmental noise. Observations and recordings were performed between 09:00 and 17:30 hrs. The insects were observed and their behavior was recorded until the first copulation or for 30 minutes if no copulations were registered. Songs were characterized and named according to the behavioral context in which they were emitted and to the previous nomenclature [26–28]: continuous wing vibration (calling phase) and intermittent wing buzz (buzzing, courtship phase). In this work, the signals during the intermittent wing buzz phase were used to compare the insects in different conditions [28]. These signals were used as they are emitted by males when they are in direct contact with females and precede copulation. Due to these characteristics, it was very clear to identify the males that produce the signal, which is considered to be decisive to males' reproductive success. In the calling phase (continuous wing vibration), usually more than one male in the arena can produce signals at the same time that are superimposed, which makes it difficult to identify the individual that produces the signal.

The acoustical signals were recorded with a professional microphone (AKG C1000S, AKG Acoustics, Nashville, USA) measuring 3.3 × 22 cm (diameter × height) and response range of 50 Hz to 20 KHz. The signals captured by the microphone were amplified by a professional amplifier Hot Sound SPA4300 (Hot Sound Indústria e Comércio de Equipamentos Eletrônicos Ltda., Valinhos, São Paulo), digitized by USB audio-captures UA-25EX (Edirol-Roland-24 bits-96 KHZ), and stored to the computer using Cool EditPro (Syntrillium software 2001, Fort Wayne, Indiana, USA) at sample rate of 46 bits and 44.100. Further analyses were done using Sound Forge 4.5 (Sonic Foundry).

**Figure 2:**
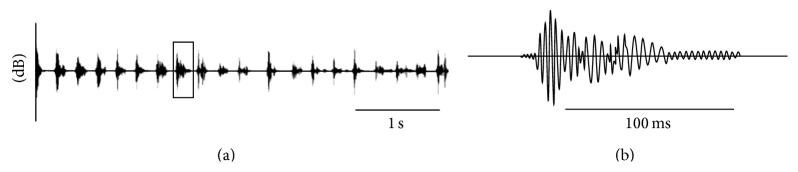
Oscillogram of one buzz signal sequence (pulse train) of* C. capitata* males (a). Details of an individual pulse (b).

Signals were characterized by calculating their temporal and spectral parameters using 30 to 50 pulses of each individual (*n* = 5–10 for each treatment). A pulse was defined as a homogeneous unitary parcel of vibration of finite duration and pulse trains as repeatable and temporally distinct groups of pulses and song as a sequence of pulses and/or pulse trains with distinct beginning and end [29]. Spectra were determined by the dominant frequency (Figure 2). Frequency spectra and sonograms were obtained by Sound Forge 4.5 software (Sonic Foundry) using the following parameters: frequency 0 to 500 HZ, FFT size 8.192, and 99% FFT overlap. Data were smoothed applying the Blackman-Harris method. Temporal characteristics of pulses are presented as pulse duration and interval between pulses (the difference in time between two consecutive pulses). Signal duration was measured between its onset and its end where the amplitude decreases within the noise level. The pulse interval was measured between onsets of one pulse and the completion of the previous pulse (Figure 2).

In order to compare the proportion of tested insects in each group (wild, sterile, and sterile treated with GRO) that emit buzz signals and the proportion of insects that successfully copulated (in relation to those that emit buzz), *χ* ^2^ test was performed. The mean pulse duration, mean interval between pulses, and mean dominant frequency for the different treatments were compared through mixed linear models using treatment as fixed effect and individuals as random effect, *P* values and Markov chain Monte Carlo confidence intervals were calculated with 10000 randomizations, and significance of fixed-effects was established with *t* statistics (*P* < 0.05). All analyses were performed using the statistical program R 3.0.1 [30].

## 3. Results

### 3.1. Geometric Morphometrics Analysis

**Figure 3:**
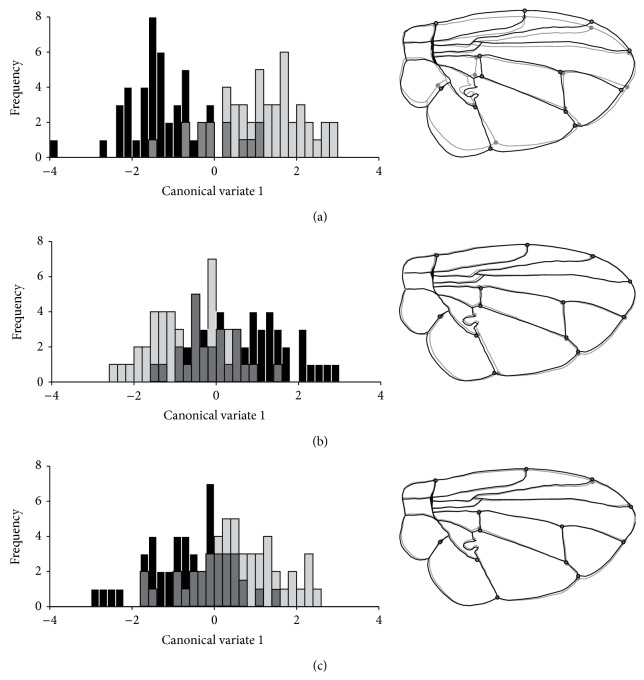
Histograms of the first canonical variate axis (CV1) for males* C. capitata* groups with (black bars and lines, accepted for copulation) and without (gray bars and lines, rejected for copulation) successful copulation using wings' shapes data, in (a) wild, (b) irradiated, and (c) irradiated and treated with ginger oil individuals. The comparison of the wings' morphology from warped outlines is at the right side of each group.

**Figure 4:**
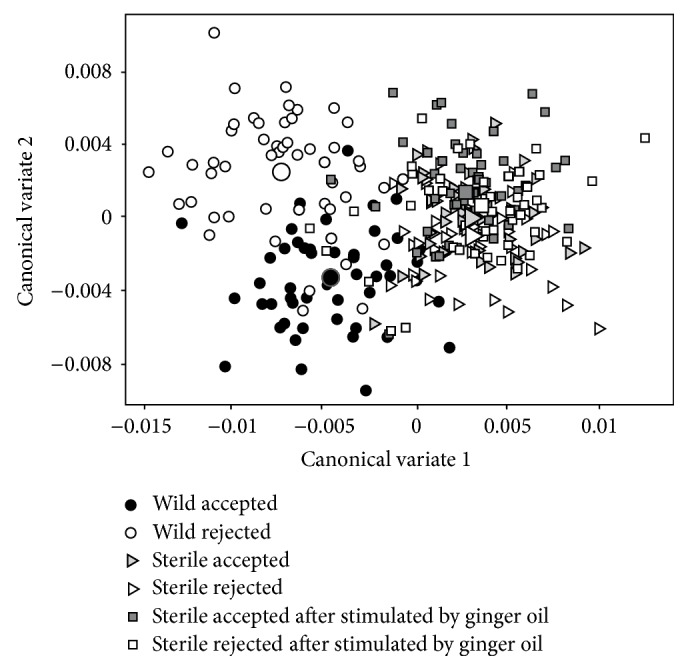
Scatter plot of* C. capitata* males as the shape of the wing along the CV1 (70.04%) and CV2 (15.79%) axes. Seconded symbols indicate the morphometric mean of each analyzed group.

The canonical variate analysis of the patterns of wings' shape in wild males of* C. capitata* revealed significant morphometric divergences (*P* < 0.01) when compared with the pattern for sterile males (Table 1, Figure 3(a)). Similarly, discriminant function analysis (DFA) between the groups indicated significant differences (*P* < 0.01). Individuals with and without successful copulation were discriminated by the medial shape of their wings in relation to CV1 axis, which explains 70.04% of the observed variation, as well as in relation to CV2, which explains 15.79% (Figure 4). Morphological differences in the wings of sterile individuals stimulated or not by ginger oil (Groups 2 and 3) were small and not significant (Table 1, Figures 3(b) and 3(c)).

Noticeably, the differences observed between the wild individuals, with and without successful copulation, involved mainly the width of the wing (landmarks 2, 3, 7, and 8), which was greater among those who succeeded in copulation. Similarly, when comparing the wing shape of wild males to that of sterile males, there was a difference related to the width and the length of the wings; that is, the sterile males had elongated and narrow wings relative to the wild ones.

Individuals with and without successful copulation were discriminated by the medial shape of their wings in relation to CV2 (Figure 4). Morphological differences in the wings of sterile individuals stimulated or not with ginger oil were small and not significant when compared to each other (Table 1, Figures 3(b) and 3(c)).

### 3.2. Acoustic Communication and Copulation Success

**Figure 5:**
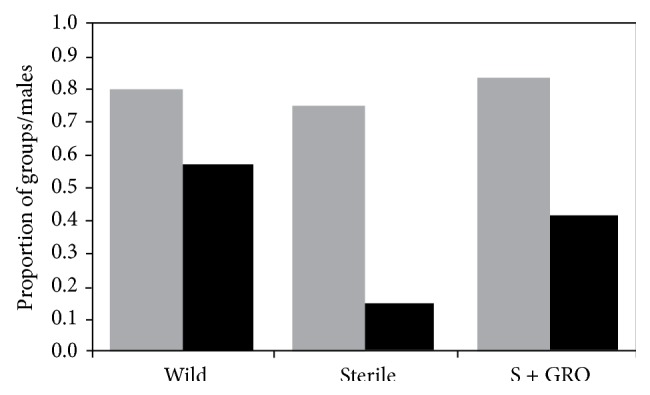
Proportion of male groups that emit buzz signals (gray bars) and proportion of males in each group that, after the emission of buzz signal, had successfully copulated (black bars), from males of three different treatments. Wild = wild population, sterile = insects irradiated in pulpal stage, and sterile + GRO = insects irradiated in pulpal stage and treated with GRO by aromatherapy. There is no statistical differences between the proportion of tested groups that emit buzz signals and significant differences between males that had successfully copulated after buzz emissions (*χ* ^2^ test *P* = 0.05).

**Figure 6:**
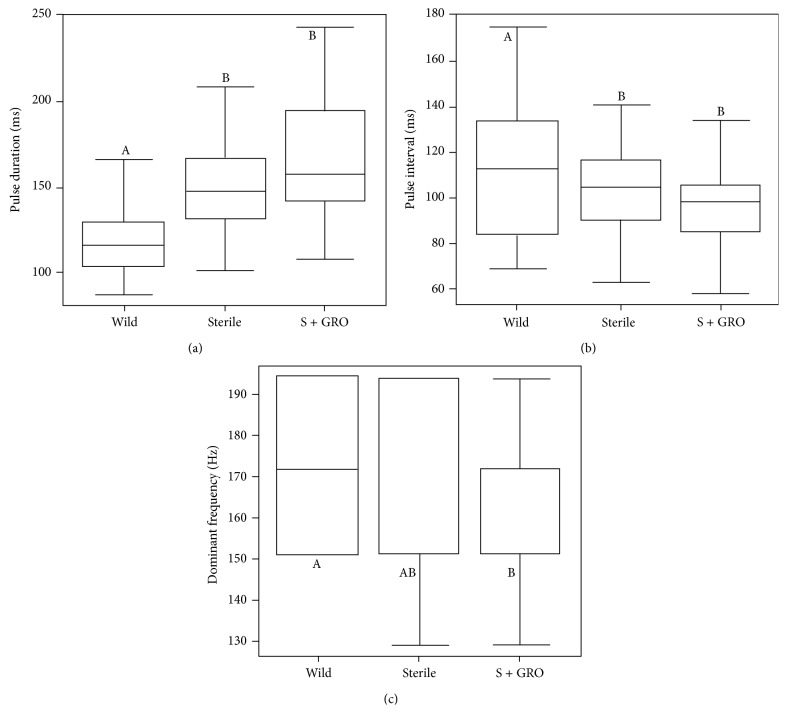
Box plots of minimum, maximum, and medium pulse duration (a), pulse interval (b) and dominant frequency (c) of* C. capitata* buzz signals of males. Boxes indicate percentages (25–75%), horizontal line in the box indicates median value, and horizontal lines out the box indicate minimum and maximum values at distances ≤1.5x from distance between percentiles. Male groups labeled with the same letter did not differ statistically (MLM and *t* test, *P* > 0.05). Wild = wild population, sterile = insects irradiated, and sterile + GRO = insects irradiated in pulpal stage and treated with GRO by aromatherapy.

Males of the three treatment groups showed the same capacity to emit buzz signals (*χ* ^2^ = 1.3; *P* = 0.52) (Figure 5). When the copulation success was compared among males groups, results showed a significant difference in the proportion of males that copulated with wild females (*χ* ^2^ = 8.6; *P* = 0.013). A higher proportion of wild males that emit buzz signals achieved copulation (Figure 5) relative to Groups 2 and 3. Males of Group 3 displayed a higher proportion of copulation than males that were not treated (Figure 5). Buzz signals showed significant differences in pulse duration, pulse interval, and dominant frequency. Wild males presented lower pulse duration, higher intervals between pulses, and higher dominant frequency (Figure 6).

## 4. Discussion

In tephritids, wings play an important role in cohort behavior. In lek, for example, males produce, release, and disperse pheromone through the vibration of wings to attract virgin females [1]. In addition to dispersing pheromone, the vibration of those males' wings also produces acoustic emission to which various functions were assigned such as calling, courtship, approximation, signalizing, premating, and copulation among others [31]. Up to the present moment, mating acoustic emissions were characterized for ten species of tephritids [32].

The success of sterile insect release programs is directly related to the success of copulation between sterile males and wild females. Changes in certain morphological characters have been frequently used to detect environmental stress in insects. The mass production of sterile insects, for many generations, can cause different types of deformations [3] that are not detected in quality control but could be factors determining successful copulation with wild females in the field.

In this study, morphological differences were found between the wings of all analyzed wild individuals (*P* < 0.01). The variation detected in the wing shape of wild males that succeeded or not in copula (MANOVA) was enough to distinguish the two groups (Figure 3). The difference between the wings of wild males accepted and nonaccepted by the females was basically in width. When comparing the wings of wild males and sterile males (treated or not with GRO), we observed that, regarding the latter, variations occur considering two aspects: height and width. Gilchrist and Crisafulli [22] studied variation in the wings of wild and mass-reared males of* Bactrocera tryoni* and found that wing shape differed between these groups. The wings of sterile males from* B. tryoni* varied significantly in length and width, compared to the wings of wild individuals from regions with high altitudes. These data are corroborated with those obtained in this work for* C. capitata*.

Changes in wing shape resulting from artificial selection under mass-rearing may affect the sexual success of the sterile males, since the results presented here show that temporal and spectral parameters of the beating of wings feature different patterns between wild and sterile males. Studies have shown that sterilization of* C. capitata* males through pupal irradiation, two days before the emergency, using 95 Gy gamma irradiation (Co-60), does not affect the copulation index with wild females compared to nonirradiated males from laboratory [33]. The process of production in large scale of males of* C. capitata per se* may affect individuals presenting malformations/morphological differentiation or even behavioral changes, making them less sexually competitive than wild males when released in the field. This can be mainly influenced by factors such as genetic drift and artificial selection in the environment where they are bred [34]. On the other hand, the exposure of sterile males of* C. capitata* to aromatization with ginger oil, which contains the attractive compound *α*-copaene, has increased the reproductive success of these males compared to nonirradiated sterile males, a fact that had been previously demonstrated by other authors [15, 33–35]. The aromatic treatment with GRO did not alter the physiological behavior of the beating of wings of sterile males during the courtship. The fact that patterns of acoustic signals emitted by these males were similar to those emitted by sterile males would indicate that the different patterns of morphometric data of the wings of wild and sterile males can be related to the observed differences in acoustic signals. Thus, the greater acceptance of sterile males treated with GRO by wild females would be related to other physiological or behavioral changes of males not considered in this work or simply be due to the more attractive odor emitted by them [35].

Additionally, the results suggest that the first canonical variable scores (CV1) can be employed to distinguish wild males of* C. capitata* from sterile males. Thus, the improvement and development of new procedures and handling for colonization and management of creations are required to minimize genetic, physiological, morphological, and behavioral wear caused by the process of sterile males' mass production. The patterns of wings obtained in this study of wild males accepted for copulation could be used in the selection of parental males of mutant strain* tsl* Vienna 8, so they were used in industrial production in biofactories for being released in the field, thus obtaining greater success in programs using the sterile insect technique for* C. capitata*.

## 5. Conclusion

The conjunction of the analyzed factors allowed a better understanding of the factors influencing the reproductive success of sterile males,* tsl* Vienna 8, with and without treatment with the aromatic ginger oil. Data suggest the usefulness of CV1 scores on morphological distinction of wild and sterile males of* C. capitata* based on the wings. The noninterference of the irradiation process as well as the aromatic treatment with GRO on the emission of buzz signals by males is clear. On the other hand, successful copulation could preferably be related to the duration and pulse interval and to the dominant frequency of the signals produced by the wings, which are probably associated to morphological changes of the wings.

## Figures and Tables

**Table 1:** Statistical tests on the morphology variation of the wing of *C*. *capitata* males.

Groups	MANOVA^∗^						DFA				
	SS	df	*F*	*P*	Pillai's trace	Pillai's trace *P*	*T* ^2^	*P*	*D* ^2^	Prc dist	*P* ^∗∗^
Wild accepted × wild rejected	0.0040	22	6.25	<0.01	0.57	<0.01	129.33	<0.01	2.29	0.013	<0.001
Steriles accepted × steriles rejected	0.0006	22	2.29	<0.01	0.29	0.15	39.03	0.15	1.26	0.005	0.007
Steriles accepted × steriles rejected (after stimulated by ginger oil)	0.0005	22	1.57	0.26	0.25	0.30	32.81	0.30	1.15	0.004	0.304
All groups	0.0270	110	11.61	<0.01	1.38	<0.01					

^∗^Only presented for data of shape. MANOVA: multivariate analysis of variance; DFA: discriminant function analysis; SS: sum square; df: degrees of freedom; *D* ^2^: Mahalanobis distance; *T* ^2^: Hotteling's test; and Prc dist: Procrustes distance; ^∗∗^Significance value to permutation tests under Procrustes distances among groups.
